# Designing an Informative App for Neurorehabilitation: A Feasibility and Satisfaction Study by Physiotherapists

**DOI:** 10.3390/healthcare11182549

**Published:** 2023-09-14

**Authors:** María Teresa Sánchez-Rodríguez, Mónica Yamile Pinzón-Bernal, Carmen Jiménez-Antona, Sofía Laguarta-Val, Patricia Sánchez-Herrera-Baeza, Pilar Fernández-González, Roberto Cano-de-la-Cuerda

**Affiliations:** 1 Department of Physical Therapy, Occupational Therapy, Rehabilitation and Physical Medicine, Facultad de Ciencias de la Salud, Universidad Rey Juan Carlos, Alcorcón 28922, Spain; mt.sanchezro@alumnos.urjc.es (M.T.S.-R.);; 2 Department of Human Movement, Universidad Autónoma de Manizales, Manizales 170002, Colombia

**Keywords:** acceptance, apps, eHealth, feasibility, neurorehabilitation, mHealth, mobile application, physiotherapists, satisfaction

## Abstract

Background: New technologies have gained popularity, especially the use of mobile phone applications, in neurorehabilitation. The aim of this paper was (1) to develop a free mobile application (*NeurorehAPP*) that provides information about and helps to select the appropriate mobile application related to a list of neurological disorders (cognitive impairment, Alzheimer’s disease, Parkinson‘s disease, multiple sclerosis, traumatic brain injury, stroke, cerebral palsy, muscular dystrophy, spina bifida, and facial paralysis), based on different objectives such as healthy habits, information, assessment, and treatment; and (2) to assess the feasibility, acceptability, and degree of satisfaction by physiotherapists after using *NeurorehAPP* for a minimum of three months. Methods: A free application was created to work with the Android^®^ operating system. The degree of satisfaction and acceptance with the application was assessed with an adaptation of the Customer Satisfaction Questionnaire through a survey via email applied to physiotherapists from hospitals and neurological rehabilitation centers in Spain after using the application. Results: *NeurorehAPP* includes a total of 131 apps. A total of 121 physiotherapists completed a satisfaction survey. The total sample showed 85.41% satisfaction with the service provided by the app and 86.41% overall satisfaction with *NeurorehAPP*. Conclusions: *NeurorehAPP* is a free, intuitive, and friendly app used with the Android^®^ operating system that allows the selection of the most appropriate app according to the type of user, neurological disorder, objective, and FDA criteria. Physiotherapists showed a high degree of satisfaction and acceptance with *NeurorehAPP*.

## 1. Introduction

According to a report published by the World Health Organization (WHO) in 2006, neurological disorders affect approximately one billion people worldwide, regardless of sex, education level, or income level [1]. Population increases and aging have resulted in greater costs and higher percentages of total disability-adjusted life years (DALYs). In this context, the prevalence of neurological diseases is estimated to reach 1136 million people by 2030 [1], so health systems will have the challenge of managing this global problem [2,3].

Information and communications technologies (ICT) are increasingly being used in healthcare, which changes traditional management strategies, especially because of the possibilities they offer in care, facilitating enhanced access to information and communication across large distances and/or monitoring subjects with neurological disorders [4,5].

The use of ICT for healthcare has been called “eHealth”. On the other hand, mHealth is described as the use of mobile devices to provide healthcare and information to consumers. mHealth uses mobile applications, or apps, to provide new perspectives for patients and health professionals [6,7]. Apps are programs developed to run on mobile devices, and they are normally adapted to the device’s processor specifications and storage capacity [8,9].

Since smartphones became popular, numerous health-related apps have been developed for different users. Health is the third-fastest-growing app category after games and general apps. The number of health-related apps is expected to increase at a rate of 23% per year over the following 5 years [10], with an increasing number of clients in the mobile app sector [10]. In view of the exponential increase in the number of health-related apps on the market, the US Food and Drug Administration (FDA) and the European Union are working towards establishing quality criteria for these apps [11,12,13]. This also applies to mobile apps potentially used in or specifically designed for neurorehabilitation [11].

This is why a mobile application, called *NeurorehAPP*, an informative app, was designed into which other mobile applications were embedded, describing their degree of compliance with the FDA criteria and suggesting various app categories (such as healthy habits, informative, assessment, treatment, and specific applications) linked to several neurological disorders to aid professionals, patients, family members, and caregivers in selecting the most appropriate app related to their needs. This integrated design enabled users to quickly access information based on their needs with a single click. Also, to date, no previous studies have designed and evaluated the feasibility and satisfaction of a mobile application in a neurorehabilitation context by the physiotherapists after its use; therefore, the relevance of this study is justified.

The aims of the present study were (1) to design and develop a free mobile application (*NeurorehAPP*), following the Food and Drug Administration (FDA) criteria, which informs about and helps to select the appropriate mobile application related to a list of neurological disorders, based on different objectives such as healthy habits, information, assessment, and treatment; and (2) to assess the feasibility, acceptability, and degree of satisfaction by physiotherapists after using it for a minimum of three months.

## 2. Materials and Methods

The research was carried out in three phases: (1) The development of *NeurorehAPP* was informed by a previously published systematic review carried out by the research group [14] in the first stage, through a bibliographic search in biomedical databases as well as in other sources of information, classifying the applications in five categories to create the content of the mobile application; (2) a free app was created for the Android^®^ operating system, using the JAVA programming language and an SQLite database, with an intuitive and friendly interface model; (3) the degree of satisfaction, acceptance, and feasibility of the app was assessed through a survey of physiotherapy professionals after a period of use.

### 2.1. Systematic Review for the Creation of the App

A previous systematic review published by our research group was used [14] for the creation of the content of the app. It included all mobile applications related to the neurorehabilitation field based on a literature review carried out in the main electronic databases [14], considering articles in English, French, and Spanish. The review included articles published from 2000 to February 2015 (the date when the research was accepted for publication), due to 2000 being documented as the year of the proliferation of health technologies in society [5]. Those manuscripts that did not focus on mobile applications specialized in neurorehabilitation before 2000 and in other languages than those indicated previously were excluded.

Additionally, a search of the mobile applications in other information sources was carried out without excluding at the beginning any application independent of its language or country, but it excluded those that could not be used in this area. The research team with expertise in the neurorehabilitation area searched mainly in app markets, reports already published on health apps, application databases, social networks, and news published in the media. For these searches, the main operating systems were taken into account: iOS, Android, BlackBerry OS, Symbian, and Windows Phone.

Once all potential applications were located, a process of selection was carried out for those that possessed potential applications in the neurorehabilitation field based on the criteria described in [14]. The different applications were categorized based on five categories so that the selected applications could be oriented to:

Healthy habits. Those applications that focus on improving patients’ lifestyles (for example, a healthy and balanced diet, good hydration, or regular practice of physical exercise).

Informative. Those whose main function is to provide complete and detailed information about a specific neurological disorder or medical aspects, regardless of whether the format is text, image, or video.

Assessment. Applications that help to perform a correct diagnosis, assessment and/or monitoring were included in this group, providing data of value to the healthcare professional.

Treatment. Those applications that might be used to treat neurological patients were included. Within this group, there will be differences between those who contemplate objectives in the physical, cognitive, and speech therapy areas.

Specific. Those apps that are specifically designed by teams and/or specific neurorehabilitation services in the approach of specific neurological disorders, based on their elaborated criteria or needs to be covered, are able to make education and active participation easy for the patient, provide necessary information for their treatment, are helpful for families, or help them find self-help groups or professionals in this field. These categories are devoted to addressing specific health disorders, including Alzheimer’s, headaches, brain injuries, muscular dystrophy, multiple sclerosis, spina bifida, stroke, and Parkinson’s, among others.

### 2.2. App Design: NeurorehAPP

Android Studio [15] version 2.0 was used as an integrated development environment (IDE) and officially created for the Android platform to create the necessary code and corresponding screens to allow users to perform a series of filters adjusting to each profile and display the applications that best fit each search criteria. *NeurorehAPP*, an informative, free mobile application for the Android operating system, was designed and released to Google Play on 5 December 2016.

The JAVA programming language was used, and based on its structure, syntax rules, programming paradigm, and object-oriented programming (OOP), as well as the functions that the language supports [16].

For the app creation, the database SQLite [17] was used, as it works in conjunction with Android applications. To integrate the database into our system, MyDbHelper was used, which was responsible for creating the connection between the databases and the generated chart for the applications. To make modifications to the database, AplicacionesDataSource [18] was used. The database was used to store the information on the applications related to the neurorehabilitation described in the previous section. The stored data were the type of user, pathology (cognitive impairment, Alzheimer’s disease, Parkinson’s disease, multiple sclerosis, traumatic brain injury, stroke, cerebral palsy, muscular dystrophy, spina bifida, and facial paralysis), classification (healthy habits, informative, assessment, treatment, and specific applications), name, operating system, price, language, release date, update, name of the developer, brief description, and FDA criteria [12,13].

The system allowed the user to access a download link for the selected application, in case that application is developed for Android, select an application as a favorite, and display the list of favorite applications. It showed the user general recommendations about the correct use of the applications in the health sciences. It will also show the user the *NeurorehAPP* developer’s profile and offer the possibility of contacting them by email.

The interface, composed of buttons, graphics, icons, and backgrounds, was designed with a friendly and different visual appearance based on different levels. The work of interface design consisted mainly of obtaining an application that, in addition to being easy to use, had visual coherence with the suitable platform.

### 2.3. Acceptance and Satisfaction Degree Assessment

To assess the acceptance and satisfaction degree with the *NeurorehAPP* application, an anonymous questionnaire was administered via email to physiotherapists. Inclusion criteria were as follows: >18 years of age, a degree in physical therapy obtained in Spain, expertise in neurorehabilitation, and the ability to work with neurological patients at hospitals or neurological rehabilitation centers in Spain. The exclusion criteria were not having completed all the data shown in the survey and not having used the app for a minimum of three months. All subjects completed the questionnaire anonymously.

To assess acceptance and satisfaction, an adaptation of the Customer Satisfaction Questionnaire (CSQ-8), designed by Roberts, Atrkisson, and Mendias in 1984, was used to evaluate briefly and generically the degree of acceptance of a given health service and to provide the opinion of the users about the services they have received. This is a brief and standardized measure of customer satisfaction that is easy to understand and quick to answer.

The modified questionnaire used (Table 1) provides information about the degree of satisfaction with the service received (section A): composed of 6 items with Likert response items with scores from 1 to 4 points, obtaining a maximum score of 24 points in the survey; and with the satisfaction degree with the mobile application (section B) in a specific way through 14 items: accessibility, easy use, graphic design, privacy and security, free character, app size and download time, clarity of information, neurological disorders included, the categories included in the app, the scientific evidence it offered, information from the apps included, apps assessment based on FDA criteria, user expectations, and general satisfaction level. Scores were set on a Likert scale of 1 to 5, with a maximum score of 70. All scores for both sections were transformed into percent (%).

All respondents were informed about the present research, and they voluntarily agreed to participate. All provided written, informed consent.

All the surveys were sent to the researchers by email. This work was approved by the Research Ethics Committee of the Rey Juan Carlos University (Ref: 010920167116).

### 2.4. Statistical Analysis

The SPSS statistical analysis program 20.0 version was used to analyze the results. We performed a descriptive analysis of the answers by range, minimum, maximum, mean, and standard deviation (SD), as well as percentage (%) reported for each item and total sections.

## 3. Results

### 3.1. Systematic Review for the Creation of the App

All data related to the previous systematic publication by the research group can be found in Sánchez-Rodríguez et al., 2017 [14]. All applications found were classified and summarized in the following categories: 17 healthy habits apps, 31 informative apps, 35 evaluation apps, 37 treatment apps (differentiating 24 for physical treatment, 7 for cognitive treatment, and 6 for the treatment of speech therapy), and 29 specific apps in neurorehabilitation. It should be noted that the same app could be aimed at several categories based on its performance. Depending on the audience to whom they were addressed, 54 applications were aimed at patients and families, 47 at health professionals, and 30 applications were for both professionals and patients and families. According to the price, 25 apps had a fixed price, while the remaining 106 were free (Supplementary Material Table S1).

### 3.2. App Design: NeurorehAPP

**Figure 1:**
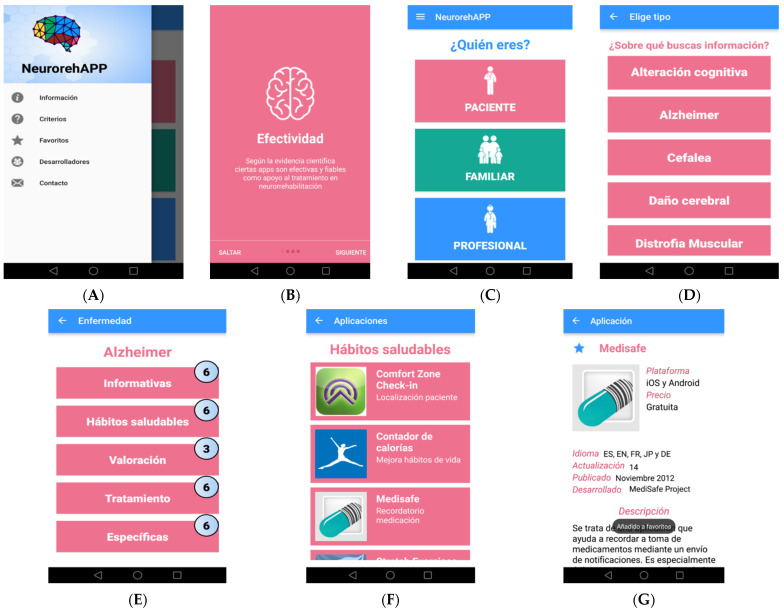
*NeurorehAPP*. (**A**) Main menu screen; (**B**) general recommendations screen; (**C**) type of user’s selection; (**D**) type of neurological disorders’ selection; (**E**) apps classification screen; (**F**) example of a list of applications’ screen; (**G**) example of information about an app included in *NeurorehAPP*.

After using the Android Studio program with the programming language JAVA and creating the database via SQLite, the *NeurorehAPP* mobile app, free for the Android operating system, was designed and released to Google Play on 5 December 2016. It is considered a search engine for mobile applications in the neurorehabilitation field, filtering information depending on the type of user and type of neurological disease (cognitive impairment, Alzheimer’s disease, Parkinson’s disease, multiple sclerosis, traumatic brain injury, stroke, cerebral palsy, muscular dystrophy, spina bifida, and facial paralysis), classification of apps based on the five specific categories, names of applications, operating systems that are available, price, language, release date, updates, developer name, short description, and FDA criteria. A model of a visual and friendly interface was designed (Figure 1A–G).

### 3.3. Assessment of the Degree of Acceptance and Satisfaction

A total of 200 surveys were sent out, and 121 were completed by physiotherapists (response rate: 60.5%). They included 39 men and 82 women, whose mean age was 29.78 years (±7.68). Descriptive statistical results concerning the scores of each section of the survey of satisfaction (satisfaction with the service received and satisfaction with the implementation of *NeurorehAPP*) are shown in Table 2.

In relation to the degree of satisfaction with the service received, a large proportion of the sample (91.7%) evaluated the quality of the app between “good” and “excellent”. Similarly, 82.7% of the sample said the app had covered their needs with a rating between “Most” and “Almost all”, and 99.2% of the sample indicated that they would use this app with a score between “Probably yes” 257 and “Definitely yes”. Overall, most of the sample showed satisfaction (85.41%) with the service provided by *NeurorehAPP* (total section A).

Concerning the degree of satisfaction with the *NeurorehAPP* application, the sample found the following as the best features of *NeurorehAPP* (>85%): accessibility, ease of use, graphic design, privacy and security, size and download time, clarity of information provided, categories of the apps included, information about scientific evidence of the apps included, technical information of the apps included, information about the evaluation of the apps included, and compliance with initial expectations. 85.9% of the sample indicated that *NeurorehAPP* had covered their initial expectations with scores between “Satisfied” and “Very Satisfied”. The overall satisfaction with *NeurorehAPP* was 86.41% (total section B).

## 4. Discussion

To our knowledge, there is no prior study to ours [14] that has rated a mobile application available with potential use in the field of neurorehabilitation. One hundred and twenty-one physiotherapists with expertise in neurorehabilitation assessed *NeurorehAPP* after a minimum of three months of use.

The results of the survey of satisfaction with the service received showed that sections A.6 (If you were looking for an app in neurorehabilitation, would you use this app?) and A.4 (If a friend needs a similar app, would you recommend it?); and thirdly, with the same score as sections A.1 and A.5 (How do you assess the quality of the app? and Has the information provided by the app helped to manage your/the problem/disease more effectively?) as the features with the highest score. 82.7% of the sample said the app had covered their needs, with a rating between “Most” and “Almost all”. In addition, they would use the application again for 92.25% of the sample. Regarding the characteristic techniques of *NeurorehAPP*, B.2, B.6, B.7, and B.9 sections were the best rated (easy to use, size and download time, clarity of the information provided, and category of included apps, respectively). The level of satisfaction with the service provided was 85.41%, and overall satisfaction with the application was 88.2%.

There are no previous satisfaction studies with which to compare our results. However, other publications have assessed the level of satisfaction after managing mobile applications in cancer [19], HIV [20], asthma [21], and other chronic diseases [22], showing similar levels of acceptance and satisfaction and even higher for *NeurorehAPP*. Compared to other mobile applications, *NeurorehAPP* boasts a significant advantage in its capacity to integrate a range of mobile applications aimed at health professionals, patients, and families/caregivers. This innovative design would enable users to identify and choose the most appropriate app, following the FDA criteria for their specific needs.

Based on the mobile applications contained in *NeurorehAPP*, there are plenty of apps in the field of neurorehabilitation, mostly related to treatment, followed by evaluation, information, and specific apps in neurorehabilitation. Most of the mobile applications in *NeurorehAPP* were aimed at patients and their families, and they are free. In our best knowledge, there are no other app repositories or publications but ours [14] to compare our data. In this regard, Xu et al. [23] performed a repository of applications focused on health based on the two main app stores, the Apple App Store and the Google Play Store, extracting detailed information from a total of 60,000 medical applications. On the other hand, there is the European Health Apps Directory [24], which makes a classification by type of app, areas of specialization (excluding neurorehabilitation), and several languages. Cohen, Nahed, and Sheth [25] conducted a review of mobile applications in the field of neurology but mainly focused on an anatomical atlas, medical imaging, and medical texts on neurological disease in app format. In Spain, a document about free apps for cognitive training and communication [8] was published with a clear interest in the field of neurological rehabilitation. However, none of the above papers focused exclusively on the neurorehabilitation field or on a classification, as previous authors had recommended [5].

Currently, more than half of Spaniards over 18 years old have a smartphone [14]. Smartphones, along with tablets, have become mobile devices valued by much of the population thanks to the possibilities offered by their portability. Current mobile devices are characterized by multi-touch interaction, convergence of functions (such as incorporating camera, video, text, and geolocation), larger screens, a virtual keyboard, and other forms of interaction such as voice [8,26]. According to data from 2014, it is estimated that in Spain, almost 4 million applications are downloaded from mobile devices a day, up from 2.7 million in 2012. The APP report 2014 [27] highlights the presence of 23 million active users of apps in Spain in 2014, up from 12 million in 2012. Such figures could be a potential resource for people already sensitized to the use of these technologies, such as subjects with neurological disorders as well as health professionals. In this context, the Internet and mobile applications are the main access points to medical information [28]. They also allow health education, contact with other professionals, the possibility of consultation, the dissemination of information, and can be used as tools to promote health. Therefore, apps could be considered powerful, viral, widespread, and easy-to-use tools in the health sciences.

In this context, our research group designed *NeurorehAPP*, a free mobile application for the Android^®^ operating system, to facilitate the selection of the most interesting mobile applications in relation to a list of neurological diseases based on FDA criteria. The apps are a valuable support tool for health professionals; some experts have even considered them the greatest technological advances of our time [7], and this fact is no stranger to professionals in the neurorehabilitation area. However, such is the number of new applications that are available every day that it is complex to select those that are useful and are adapted to the needs of patients with neurological disorders. That is why, as the APP report points out [27], it is necessary to have new documents and/or tools to facilitate their choice in the growth of that offer. In this context, we launched *NeurorehAPP*, which aims to provide an informative application in the field of neurorehabilitation to help, according to the type of user (neurological patient, family or caregivers, and health professionals), identify applications of interest in this area based on classification categories (healthy habits, informative, assessment, treatment, and specific applications) and depending on the type of neurological disorder. Once all potential applications were located, a suggested process of mobile application selection was carried out based on the FDA criteria, with a high level of satisfaction and acceptance by physiotherapists after a minimum of three months of using it.

The FDA recognizes the wide variety of mobile applications, their rapid pace of innovation, and their potential benefits and risks to public health. In this regard, it has published papers on how they intend to apply the regulation of these applications [12], as it must monitor their safety and efficacy [13,29]. In this line, all the apps contained in *NeurorehAPP* follow these recommendations from the FDA, as well as those of Meulendijk et al. [30], based on nine essential criteria: accessibility, verifiability, portability, privacy, application security, user security, stability, reliability, and ease of use, scored and displayed in a user-friendly interface.

Our study presents several limitations. First, it is possible that, given the wealth of mobile applications and their rapid expansion, many apps could have been left out, so it is necessary to make updates to *NeurorehAPP* periodically. Second, it is important to note that all the information contained in *NeurorehAPP* will never be a substitute for professional healthcare. Further, our mobile application does not include information about the application of optical technology in the field of health care [31] or other technologies added [32], so future designs should consider these aspects. Third, although our app could be useful in addressing certain neurological diseases, its use would not always be applicable in cases where there is a cognitive impairment that hinders the understanding of the guidelines set by the application itself or in patients with motor deficits that limit the interaction with the app. Fourth, it would be interesting to assess the degree of satisfaction of patients after using *NeurorehAPP* and other health professionals related to neurorehabilitation. Finally, we do not incorporate the feedback of the physiotherapists who did not use the app for at least three months and/or disliked the mobile application. Furthermore, future studies should be conducted incorporating follow-up interviews (focus groups) to ask for feedback on barriers to adoption, among other things.

## 5. Conclusions

*NeurorehAPP* is a free app for the Android^®^ operating system that is intuitive and has a friendly interface that allows the selection of the most suitable apps in relation to a list of neurological diseases according to five categories (healthy habits, information, assessment, treatment, and specifics) and based on FDA criteria. Physiotherapy professionals with expertise in the neurorehabilitation area who evaluated the app showed a high level of satisfaction and acceptance after a minimum of three months of using it.

## Figures and Tables

**Table 1:** Satisfaction and satisfaction degree assessment.

**Section A: Satisfaction with the Service Received.**							
	**Ratings**						
	**1**	**2**	**3**		**4**		
A1. How do you assess the quality of the app?	Poor	Fair	Good		Excellent		
A2. Did you get the kind of service you wanted?	Definitely not	No, not much	Yes, generally		Definitely yes		
A3. To what extent has this app covered your needs?	None	Only a few	Most of them		Almost all		
A4. If a friend needs a similar app, would you recommend it?	Definitely not	No, not much	Yes, generally		Definitely yes		
A5. Has the information provided by the app helped manage your/the problem/disease more effectively?	No, my problem worsened	No, they have not really helped me	Yes, they have helped me more or less		Yes, they have helped me a lot		
A6. If you were looking for an app in neurorehabilitation, would you use this app?	Definitely not	Probably not	Probably yes		Definitely yes		
**Section B: Degree of Satisfaction with *NeurorehAPP***							
Now, answer the following questions. Please rate from 1 to 5 the following attributes of the app.							
**Attributes of the App**			**Score**				
B1. Accessibility			1	2	3	4	5
B2. Ease of use			1	2	3	4	5
B3. Graphic design			1	2	3	4	5
B4. Privacy and security			1	2	3	4	5
B5. Free app			1	2	3	4	5
B6. Size and download time			1	2	3	4	5
B7. Clarity of the information provided			1	2	3	4	5
B8. Diseases included			1	2	3	4	5
B9. Category of the apps included (healthy habits, informative, assessment, evaluation and specific)			1	2	3	4	5
B10. Scientific evidence information of the apps included			1	2	3	4	5
B11. Technical information of the apps included in summary tables			1	2	3	4	5
B12. Information about the evaluation of the apps included			1	2	3	4	5
B13. Fulfillment of initial expectations			1	2	3	4	5
B14. Level of satisfaction with the app			1	2	3	4	5

1 = Dissatisfied; 2 = Not satisfied; 3 = Indifferent; 4 = Satisfied; 5 = Very satisfied.

**Table 2:** Descriptive results regarding the satisfaction survey.

Questions	N	Range	Minimum	Maximum	Mean	Standard Deviation	%
A1. How do you assess the quality of the app?	121	1	3	4	3.44	0.498	86%
A2. Did you get the kind of service you wanted?	121	2	2	4	3.25	0.537	81.25%
A3. To what extent has this app covered your needs?	121	3	1	4	3.07	0.655	76.75%
A4. If a friend needs a similar app, would you recommend it?	121	2	2	4	3.61	0.506	90.25%
A5. Has the information provided by the app helped manage your/the problem/disease more effectively?	121	2	2	4	3.44	0.576	86%
A6. If you were looking for an app in neurorehabilitation, would you use this app?	121	2	2	4	3.69	0.484	92.25%
B1. Accessibility	121	4	1	5	4.31	0.973	86.2%
B2. Ease of use	121	3	2	5	4.54	0.578	90.8%
B3. Graphic design	121	3	2	5	4.35	0.629	87%
B4. Privacy and security	121	4	1	5	4.33	0.757	86.6%
B5. Free app	121	4	1	5	4.10	0.970	82%
B6. Size and download time	121	4	1	5	4.42	0.793	88.4%
B7. Clarity of the information provided	121	3	2	5	4.45	0.632	89%
B8. Diseases included	121	3	2	5	3.85	0.901	77%
B9. Category of the apps included (healthy habits, informative, assessment, evaluation and specific)	121	2	3	5	4.45	0.605	89%
B10. Scientific evidence information of the apps included	121	3	2	5	4.31	0.707	86.2%
B11. Technical information of the apps included in summary tables	121	3	2	5	4.28	0.755	85.6%
B12. Information about the evaluation of the apps included	121	3	2	5	4.41	0.691	88.2%
B13. Fulfillment of initial expectations	121	3	2	5	4.28	0.755	85.6%
B14. Level of satisfaction with the app	121	3	2	5	4.41	0.715	88.2%

## Data Availability

The data presented in this study are available upon request from the corresponding author.

## Supplementary Materials

The following supporting information can be downloaded at: https://www.mdpi.com/article/10.3390/healthcare11182549/s1, Table S1: List of apps included in *NeurorehAPP*.

## Abbreviations

FDA	Food and Drug Administration
WHO	World Health Organization
DALY	Total disability-adjusted life years
ICT	Information and communications technologies
PwC	PriceWaterhouseCoopers
CSQ-8	Customer Satisfaction Questionnaire
