# Supplementary material for: Designing an Informative App for Neurorehabilitation: A Feasibility and Satisfaction Study by Physiotherapists
Source: Healthcare (Basel). 2023 Sep 14;11(18):2549. doi: 10.3390/healthcare11182549 (PMC10530788; doi:10.3390/healthcare11182549)
Supplement: Supplementary file 1 [file healthcare-11-02549-s001.zip › healthcare-2544162-supplementary.pdf]

Table S1. List of apps with application in neurorehabilitation.

Application Name	Patient / Professional	Payment / Free	Short description	Classification	Pathology
AbaPlanet	Patients, relatives and professionals	Free	Improves communication	Treatment (Speech)	Cognitive impairment
Ablah	Patients and relatives	Paid	Improves communication	Treatment (Speech)	Cognitive impairment
Aemps CIMA	Professionals	Free	Prescribing information	Informative	All diseases
Alzheimer	Patients and relatives	Free	Information about the disease	Specific	Alzheimer
Alzeimer app	Patients and relatives	Free	Treatment and understanding of pathology	Specific	Alzheimer
Alzheimer App for Caregivers	Patients and relatives	Free	Information about the disease	Specific	Alzheimer
Alzheimer's Daily Companion	Patients, relatives and professionals	Free	Information about the disease	Specific	Alzheimer
Alzheimer Info	Patients and	Free	Information about	Specific	Alzheimer

	relatives		the disease		
Alzheimer Info & Exercises	Patients and relatives	Free	Information about the disease	Specific and Treatment	Alzheimer
AMiAlcance	Patients and relatives	Free	Improving the use of technology	Healthy habits.	cognitive impairment
Autism. Discover emotions	Patients, relatives and professionals	Free	Improving communication	Treatment	cognitive impairment
Autismo iHelp	Patients, relatives and professionals	Free	Improving communication	Treatment (Speech)	cognitive impairment
Baby Exercises and Activities	Patients, relatives and professionals	Free	Exercises for pediatric stimulation	Informative and treatment	Global developmental delay
Baby's Motor Milestores	Patients, relatives and professionals	Free	Milestones of baby motor development	Informative	Global developmental delay
BabySparks	Patients, relatives and professionals	Free	Milestones of baby motor development and exercises	Informative and treatment	Global developmental delay
Beats Medical	Patients, relatives and professionals	Free	Mobility treatment, skills and speech	Assessment and treatment	Parkinson

Drinking water (Water your body)	Patients and relatives	Free	Improving health habits (hydration)	Healthy habits	All diseases
Brainyapp	Patients and relatives	Free	Memory training	Treatment (Cognitive)	All diseases
Cath my pain	Patients and relatives	Free	Pain´s diary	Assessment	All diseases
CareZone	Professionals	Free	Prescribing information	Informative	All diseases
Cerebral Palsy	Patients and relatives	Free	Treatment and understanding of the disease	Specific	Brain Damage
Clinometer	Professionals	Paid	Work tool	Assessment	All diseases
CNS Mobile	Patients, relatives and professionals	Free	Treatment and understanding of the disease	Specific	Brain Damage
ICTUS Code	Patients and relatives	Free	Information about the disease	Specific	Stroke
Comfort Zone Check-in	Patients and relatives	Free	Patient´s location	Healthy habits	All diseases
Calorie Counter	Patients and	Free	Improving healthy	Healthy habits	All diseases

	relatives		habits (Food)		
Control EM	Patients and relatives	Free	Information about the disease	Specific	Multiple sclerosis
Daño Cerebral (Brain Damage)	Patients, relatives and professionals	Free	Treatment and understanding the disease	Specific	Brain Damage
Dexteria.Fine motor skills development	Patients, relatives and professionals	Paid	Activities for hand treatment	Informative	All diseases
DMD guide	Patients, relatives and professionals	Free	Treatment and understanding the disease	Specific	Muscular dystrophy
Dr. Goniometer	Professionals	Paid	Work tool	Assessment	All diseases
EDSS Calculator	Professionals	Paid (iOS) Free (Android)	Work tool	Assessment	All diseases
El círculo de la Salud (The Circle of Health)	Patients and relatives	Free	Improved lifestyle	Healthy habits.	All diseases
MS All in One	Patients, relatives and professionals	Free	Information about MS	Specific	Multiple sclerosis
Endomondo	Patients and	Free	Improving healthy	Healthy habits.	All diseases

	relatives		habits (exercise)		
Epocrates	Professionals	Free	Medical Informative	Informative	All diseases
Glasgow Coma Scale	Professionals	Free	Work tool	Assessment	All diseases
Stroke Scale NIHSS	Professionals	Paid	Work tool	Assessment	Stroke
Escala RACE (RACE scale)	Professionals	Free	Work tool	Assessment	Stroke
Esclerosis múltiple @Point de Care TM (Multiple Sclerosis @Point de Care TM)	Professionals	Free	Information about the disease	Specific	Multiple sclerosis
Escoliómetro HD (scoliometer HD)	Professionals	Paid	Work tools	Assessment	All diseases
Estimulación Temprana para Bebés (Early Stimulation Baby)	Patients and relatives	Free	Useful information for the baby's development	Informative	Global developmental delay
EVA Facial Mouse	Patients and relatives	Free	Helps the patient	Healthy lifestyles and treatment	All diseases
Fisioterapia a tu alcance (Physiotherapy at your fingertips)	Patients, relatives and professionals	Free	Information	Informative and treatment (Physical)	All diseases

Fisioterapia FF (physiotherapy FF)	Professionals	Free	Information	Informative	All diseases
Fisioterapia Guía de ayuda (Physiotherapy Help Guide)	Professionals	Free	Information	Informative	All diseases
Fisioterapia respiratoria (Respiratory physiotherapy)	Professionals	Paid	Work tool	Assessment	All diseases
Fit Brains Trainer	Patients and relatives	Free	Cognitive training	Treatment	All diseases
Goniometer Records	Professionals	Free	Work tools	Assessment	All diseases
Goniómetro Pro Gratuito (Free Pro Goniometer)	Professionals	Free	Work tools	Assessment	All diseases
Google Fit	Patients and relatives	Free	Improving healthy habits (exercise)	Healthy habits	All diseases
Guía de actos fisioterápicos (Guide of Physiotherapeutic Acts)	Patients, relatives and professionals	Free	Information	Informative and treatment (Physical)	All diseases
Guía salud (health guide)	Professionals	Free	Information	Informative	All diseases
Headache Diary	Patients and relatives	Paid	Pain´s diary	Assessment	Headache or migraine
Human Anatomy Atlas 3D.	Professionals	Free	Work tools	Informative	All diseases

Visible body					
ICTUS	Patients and relatives	Free	Information about the disease	Specific	Stroke
ICTUS care	Patients and relatives	Free	Treatment and understanding the disease	Specific	Stroke
IDoctus	Professionals	Free	Work tools	Informative	All diseases
INeuro	Professionals	Paid	Information	Informative	All diseases
IPediatric	Patients and relatives	Paid	Information	Informative	All diseases
ISecuencias	Patients, relatives and professionals	Paid	Improving living habits (learning)	Healthy habits.	All diseases
Kinedu	Patients, relatives and professionals	Free	Early stimulation	Treatment	Global developmental delay
LetMeTalk: Talker SAAC, CAA, SAC	Patients and relatives	Free	Improves communication	Treatment (Speech)	cognitive impairment
Lift Pulse	Patients and relatives	Free	Motion data recording	Assessment	Parkinson
To Do Lists	Patients and	Paid	Improved lifestyle	Healthy habits.	All diseases

	relatives				
ListenMee App	Patients and relatives	Paid	Improved motion by auditory cues	Treatment	Parkinson
Medical tols	Professionals	Free	Work tools	Assessment	All diseases
Medisafe	Patients and relatives	Free	Medication reminder	Healthy habits.	All diseases
Medscape	Professionals	Free	Work tools	Informative	All diseases
Mememtum	Patients, relatives and professionals	Paid	Work tools	Assessment and Treatment(Physic al)	Parkinson
Memento	Patients and relatives	Free	Working memory	Treatment (Cognitive)	cognitive impairment
Metrónomo (Metronome)	Patients, relatives and professionals	Free	Work tools	Treatment	Parkinson
Migrapp	Patients and relatives	Free	Pain´s diary	Assessment	Headache or migraine
MindMate	Patients, relatives and professionals	Free	Information and exercises	Informative and Treatment	Alzheimer and dementia
Mirror Therapy	Patients, relatives and professionals	Paid	Mirror Therapy	Treatment	All diseases

Mobile MIM	Professionals	Free	Work tools	Assessment	All diseases
MSAA Self Care Manager	Patients and relatives	Free	Information about the disease	Specific	Multiple sclerosis
MS Diagnosis	Professionals	Free	Assessment Tool	Assessment	Multiple sclerosis
MS Diagnosis and Management	Patients, relatives and professionals	Free	Information and exercises	specific and treatment	Multiple sclerosis
Multiple Sclerosis	Patients, relatives and professionals	Free	Information and exercises for MS patients	specific and treatment	Multiple sclerosis
Muscle Premium	Professionals	Free	Work tools	Informative	All diseases
Muscular Dystrophy	Patients and relatives	Free	Treatment and understanding the disease	Specific	Muscular dystrophy
Muscular Dystrophy Info	Patients and relatives	Free	Information about the disease	Specific	Muscular dystrophy
MyMigraines	Patients and relatives	Free	Work tools	Assessment	Migraines
MyPhysio	Professionals	Free	Information	Treatment	All diseases
My Epilepsy Diary	Patients and	Free	Pain´s diary	Assessment	All diseases

	relatives				
My Pain Diary	Patients and relatives	Paid	Pain´s diary	Assessment	All diseases
Neuro Attention	Patients and relatives	Free	Memory treatment	Treatment (Cognitive)	All diseases
Neuro localizar (Neuro Locate)	Patients, relatives and professionals	Free	Information	Informative	All diseases
Neuro Rehab	Professionals	Free	Information	Informative	All diseases
Neuro12	Professionals	Free	Work tools	Informative and assessment	All diseases
NeuroMind	Professionals	Free	Work tools	Informative and assessment	All diseases
NeuroNation- brain exercise	Patients and relatives	Free	Working memory	Treatment (Cognitive)	All diseases
NeuroScores	Professionals	Free	Work tools	Assessment	All diseases
Nicke + Running	Patients and relatives	Free	Improving lifestyle	Healthy habits.	All diseases
Occupational Therapy	Professionals	Free	Therapeutic activities	Informative and Treatment	All diseases
Osirix	Professionals	Paid	Work tools	Assessment	All diseases

Padmed	Professionals	Free	Work tools	Informative	All diseases
Paralisia (facial paralysis)	Patients and relatives	Free	Treatment and understanding the disease	Specific	facial paralysis
Parent Project Muscular Dystrophy (PPMD)	Patients, relatives and professionals	Free	Information about the disease	Specific	Muscular dystrophy
Parkinson Disease	Patients, relatives and professionals	Free	Research information	Specific	Parkinson
Parkinson exercises	Patients, relatives and professionals	Paid	Treatment and understanding the disease	Specific	Parkinson
Parkinson's Central	Patients and relatives	Free	Information about the disease	Specific	Parkinson
Parkinson's Toolkit	Patients, relatives and professionals	Free	Treatment and understanding the disease	Specific	Parkinson
PhysioAdvisor Exercises	Professionals	Paid	Information	Treatment	All diseases
Physotherapist	Patients, relatives and professionals	Free	Information and exercises	Treatment	All diseases
Physiotherapy Exercises	Professionals	Free	Work tools	Informative and	All diseases

				treatment (Physical)	
Physiotherapy Help Guide	Professionals	Free	Information and exercises	Assessment and treatment	All diseases
Picto Connection	Patients and relatives	Free	Improving lifestyle	Treatment and healthy habits	All diseases
Pedometer	Patients and relatives	Free	Healthy habits (exercise)	Healthy habits	All diseases
Posture: Screen mobile	Professionals	Paid	Work tools	Assessment	All diseases
Prognosis	Professionals	Free	Information	Informative	All diseases
Rancho Gait	Professionals	Free	Work tools	Assessment	All diseases
Recognise Hands	Professionals	Paid	Work tools	Treatment	All diseases
Rehab TherX	Professionals	Paid	Information	Treatment	All diseases
Rehabilitation for Lower Limbs	Professionals	Free	Information	Treatment	All diseases
SAAC. Communication. Autism.	Patients and relatives	Free	Improving healthy habits (Communication)	Treatment (Speech)	Cognitive impairment
Sophie's Drawings	Patients and relatives	Paid	Fine motor skills exercises	Treatment	Global developmental

					delay
Spaced Retrieval Therapy	Patients and relatives	Paid	Memory exercises	Treatment (Cognitive)	cognitive impairment
Spina Bífida	Patients and relatives	Free	Treatment and understanding the disease	Specific	Bifida spine
Stretch Exercises	Patients and relatives	Free	Improving healthy habits (exercise)	Informative and healthy habits	All diseases
StretchingE	Patients and relatives	Free	Improving healthy habits (exercise)	Informative and healthy habits	All diseases
Stroke Patient	Patients and relatives	Free	Information about the disease	Specific	Brain Damage
Testes Ortopédicos	Professionals	Free	orthopedic tests	Assessment	All diseases
TF: Light Table	Professionals	Free	Work tools	Assessment	All diseases
Timer +	Professionals	Free	Work tools	Assessment	All diseases
Tweri	Patients and relatives	Free	Support for localization	Healthy habits	All diseases
Universal Doctor Speaker	Patients, relatives and professionals	Free	Medical translator	Assessment	All diseases
Welvi	Patients and	Free	Improving lifestyle	Healthy habits	All diseases

	relatives				
WonderMon- Manequin	Professionals	Free	Work tools	Assessment	All diseases
3D Brain	Professionals	Free	Information	Informative	All diseases

Applications marked with gray are those belonging to the literature search databases.

Applications in white have been obtained from other sources.
